# Vaccine hesitancy and trust in sub-Saharan Africa

**DOI:** 10.1038/s41598-024-61205-0

**Published:** 2024-05-13

**Authors:** Kerstin Unfried, Jan Priebe

**Affiliations:** 1 Health Economics Research Group, Bernhard Nocht Institute for Tropical Medicine (BNITM), Hamburg, Germany; 2 Hamburg Center for Health Economics (HCHE), Hamburg, Germany

**Keywords:** Applied mathematics, Information technology, Scientific data

## Abstract

Lack of trust is a primary reason behind the global rise in vaccine hesitancy. Existing research on the trust—vaccine hesitancy nexus has almost exclusively focused on COVID-19 with the vast majority of studies examining industrialized countries. In this study, we investigated the influence of trust in different policy-relevant actors (government, science, media, pharmaceutical companies, society) on vaccine hesitancy for recently available vaccines related to polio and HPV which we benchmark against a COVID-19 vaccine. Leveraging unique primary data on 5203 individuals from six countries (Ghana, Kenya, Nigeria, South Africa, Tanzania, and Uganda), we showed that individuals’ trust in the government and society are key predictors of vaccine hesitancy. Furthermore, we demonstrated that these relationships are remarkably stable across vaccine, disease, and country contexts.

## Introduction

Immunization is one of the most cost-effective public health interventions and has helped save millions of lives globally. Despite many success stories, the progress made in tackling vaccine-preventable diseases has in many settings come under threat by increases in vaccine hesitancy, i.e. the reluctance or refusal to vaccinate despite the availability of vaccines^1^. As such vaccine hesitancy can lead to the prolongation and resurgence of vaccine-preventable diseases and cause substantial negative social and economic consequences. It is estimated that annually around 1.5 million deaths could be avoided globally if vaccination rates were higher^2,3^.

Vaccine hesitancy is primarily a trust issue^4,5^. Especially in contexts of high uncertainty, trust in pivotal institutions is a common heuristic used to shortcut decision-making with incomplete information. Systematic and literature reviews have documented a negative relationship between trust in institutions that are involved in the production, supply, distribution, and monitoring of vaccines and vaccine hesitancy^6,7^. Specifically, vaccine hesitancy has been related to levels of trust in the national and local government^5,8,9^, manufacturers/pharmaceutical firms^9,10^, healthcare systems including physicians^10–13^, and science^4,12,14,15^.

Understanding the nexus between trust and vaccine hesitancy matters for policy making as it helps to design optimal information campaigns and distribution of vaccines, among others. Despite its importance, there is a notable knowledge gap with respect to low- and middle-income countries (LMICs) in which most vaccine-preventable deaths occur. Existing research has almost exclusively focused on (i) richer countries or (ii) COVID-19 vaccines. This gap is worrisome given that existing evidence suggests that vaccine hesitancy is highly context-specific, varying across place, time, and vaccine type^14,16–26^.

The objective of this study is to fill this gap by analyzing the relationship between trust and vaccine hesitancy in the context of six sub-Saharan African countries with respect to vaccines for polio, HPV, and COVID-19. In early 2023 we gathered primary data via online surveys in Ghana, Kenya, Nigeria, South Africa, Tanzania, Uganda. In total, we collected information from 5203 adults on vaccine hesitancy concerning three types of vaccines (polio (nOPV2); HPV (GARDASIL4, CERVARIX); COVID-19 (COMIRANTY)) and trust in five distinct types of institutions (government, media, science, pharmaceutical companies, and society). The selection of vaccines was based on the countries’ national strategies to integrate these vaccines into their (standard) immunization programs. Employing a multivariate regression framework, we estimated in this paper to what extend institution-specific trust influences vaccine-specific vaccination intentions. Importantly, our study design allows to explicitly compare trust- and vaccine-specific factors, enabling us to gauge to what extent results obtained from the abundant research on COVID-19 vaccine hesitancy can be transferred to other disease and vaccine contexts.

The focus of our study on sub-Saharan Africa (SSA) was motivated by three considerations. First, vaccination and vaccine acceptance rates in SSA are particularly low in international comparisons^14,24,27,28^, and have witnessed a substantial decline in recent years^29–31^. Second, SSA represents a cultural and historical context that differs from other world regions in important ways as various controversies concerning vaccinations and trust are SSA-specific. For instance, widespread rumours exist that SSA is a testing ground for new vaccines and that Africans are used as guinea pigs in vaccine trials^32^. These rumours have contributed to vaccine hesitancy^27,33,34^. In this context, colonial medical experimentation in SSA has been found to still diminish trust into modern medicine, today^35^. Third, despite most of the world’s vaccine-preventable deaths occurring in SSA, issues of trust and vaccine hesitancy appear to be understudied for SSA^36^ with existing quantitative studies being confined to COVID-19 and a limited set of trust indicators^37–39^. Beyond COVID-19 vaccines, evidence on vaccine hesitancy in SSA primarily stems from correlational evidence related to vaccination rates but not to measures of vaccine hesitancy^8^ or from small-scale qualitative studies^40,41^.

Our study also speaks to the literature on the role of societal trust for vaccine hesitancy. With the existing literature focusing on individual trust factors, societal aspects of trust in vaccination are often neglected or disregarded^14^. Apart from individual health benefits, vaccinations create beneficial externalities that help achieve herd immunity; health benefits to other non-vaccinated members of society. The economic & psychology literature has highlighted that decisions to contribute to a collective good (vaccinations) depends on an individual’s expectations about the cooperation of other society members and an individual’s trust in others^42,43^. Hence, individual trust and beliefs about vaccination decisions of others can play a relevant role in overcoming vaccine hesitancy. By examining individual trust towards vaccination decisions of other society members, we add to the literature investigating the role of prosocial motivations in health preventive behavior^44–46^.

## Results

### Sample description, vaccination status and vaccine hesitancy

The study sample comprised 5203 individuals living in six SSA countries (Ghana, Kenya, Nigeria, South Africa, Tanzania, Uganda). The number of respondents per country is presented in Supplementary Table B1 in Appendix B. The age of respondents ranged from 18 to 75 years, with a mean of 29.15 and a median age of 27 years. About 65% of respondents were male and 35% were female. As common in online samples, respondents tended to be well-educated (about 73% possess tertiary education). Supplementary Table B2 in Appendix B provides additional summary statistics describing the socio-demographic characteristics of our total sample and Supplementary Table B3 in Appendix B reports the descriptive statistics by country.

**Figure 1:**
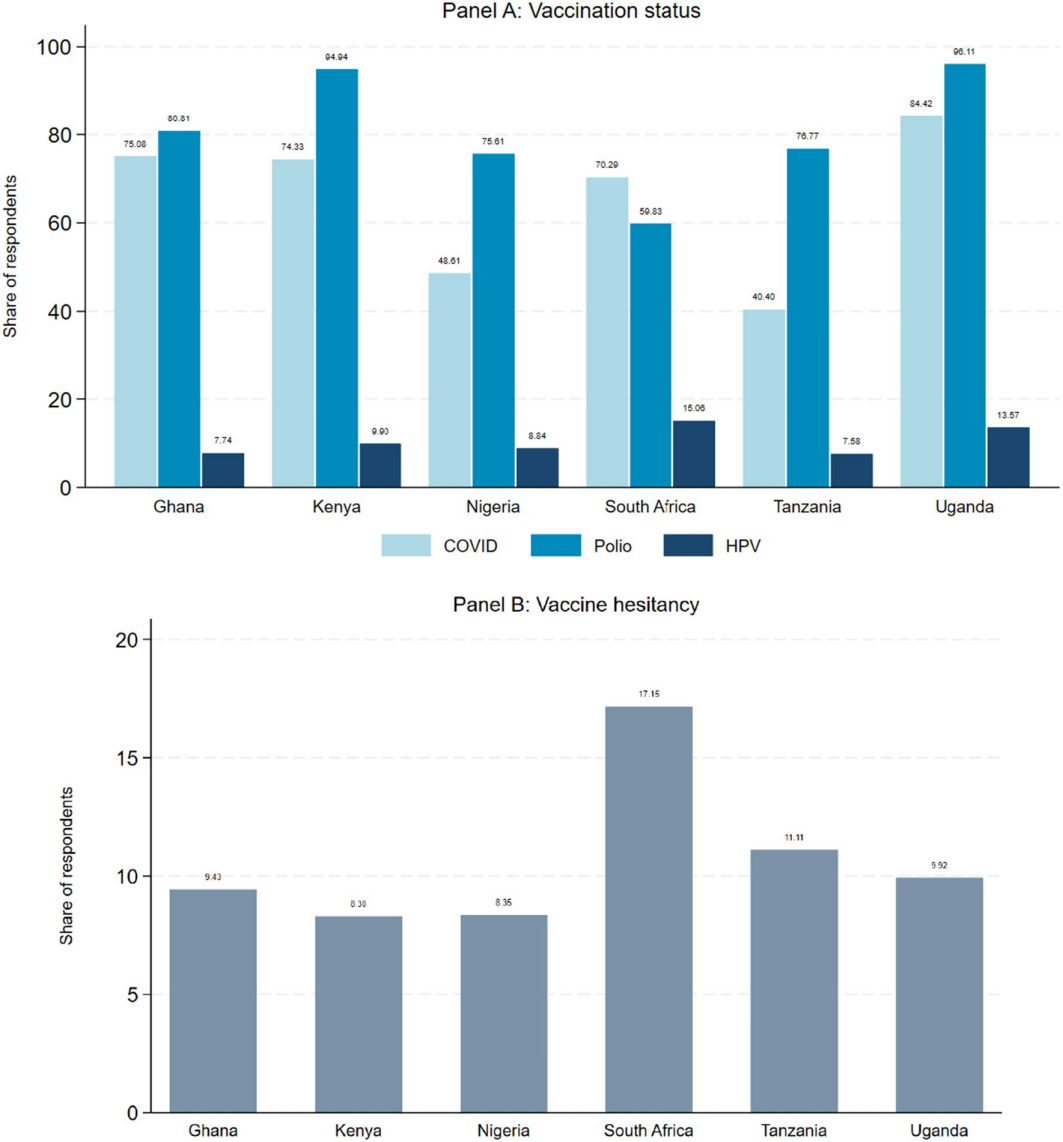
Vaccination status and vaccine hesitancy. The Figure presents the percentage of participants that reported to have received at least one vaccine against the respective disease (vaccine status) and vaccine hesitancy rates by country. Panel (**A**) presents the average vaccination status for COVID-19, polio, and HPV based on the self-reported answers in the study sample. Panel (**B**) depicts vaccine hesitancy rates measured by participants’ intention to get vaccinated with a new vaccine (COVID-19, polio, HPV).

We start with discussing the vaccination status and vaccine hesitancy rates among our survey respondents. Panel A of Fig. 1 depicts the percentage of study participants that were vaccinated against COVID-19, polio, and HPV by country. The numbers are based on individuals’ self-reports and capture whether respondents received at least one vaccination per disease. A majority of respondents stated that they were vaccinated against polio (89.74%) and COVID-19 (71.42%), while a minority said that they received any vaccination against HPV (about 10%). The national vaccination rates based on statistics from WHO, UNICEF, and national reports are reported in Supplementary Table B4 in Appendix B. Comparing these official statistics to the self-reported vaccination status in our sample, we see that generally vaccination figures match quite well. The vaccination status for COVID-19 was higher compared to the published official vaccination rates in the respective countries. We believe that this is due to the circumstance that respondents in our sample were better educated and predominantly resided in urban areas. Additionally, the rate of vaccinated persons against HPV are lower in our study sample compared to the national statistics that might be explained by the difference in the underlying population sample.

Furthermore, Fig. 1 shows considerable variations across countries and disease. Vaccination status were highest in Kenya and Uganda, and lowest in Nigeria, South Africa and Tanzania.

Panel B of Fig. 1 depicts the share of vaccine-hesitant respondents by country. Vaccine hesitancy was defined as (i) having no intention to get vaccinated (if the person is not yet vaccinated) or (ii) having no intention to recommend the vaccination to other family members (if respondent was already vaccinated against the disease). [Likewise, the second part of the question allowed us to measure vaccination attitudes also in cases in which vaccinations are mainly recommended for females (HPV) or children (polio).] About 9% of our sample were classified as vaccine hesitant. Vaccine hesitancy differed substantially across countries and ranged between 8.3% in Kenya and 17.15% in South Africa.

### Trust and vaccine hesitancy

**Figure 2:**
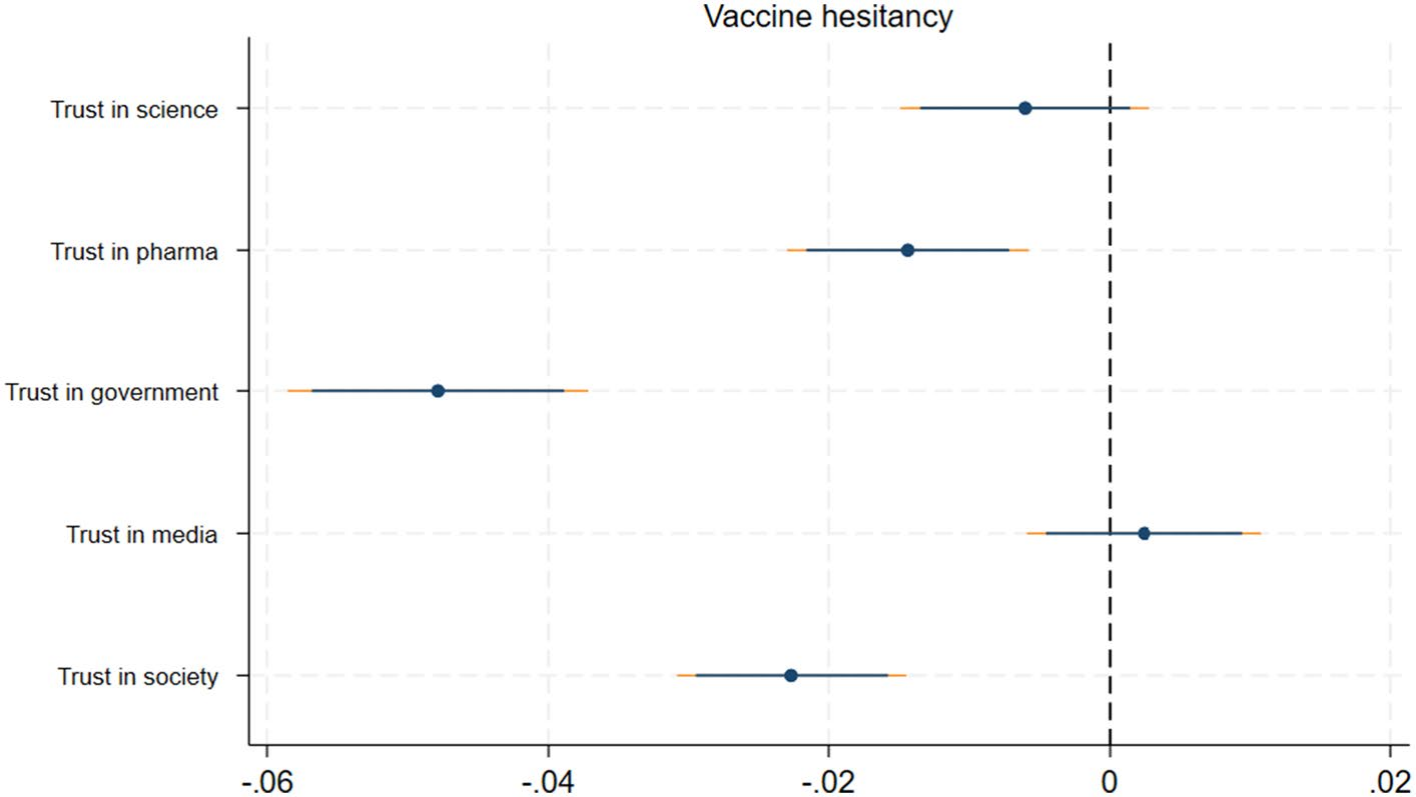
Trust and vaccine hesitancy. The Figure reports coefficient estimates and confidence intervals at the 95 and 90 percent level. Results are obtained by OLS. Controls included are age, gender, marital and employment status, wealth, education, religion, personality traits, and vaccination history as well as country and vaccine-type fixed effects. Robust standard errors are used.

Employing linear probability models—as described in section ’Empirical strategy”—we estimated by ordinary least squares (OLS) how individuals’ level of trust affects vaccine hesitancy. The adopted regression framework controlled for a number of individual and country-specific characteristics as well as vaccine type. As explanatory variables we considered trust in science, pharmaceutical firms, the government, media, and society. These variables captured respondents’ level of trust into the respective institutions with regard to health matters. We proxied trust in society from a behavioral perspective; respondents’ beliefs about the extent to which other members of society intend to get vaccinated with the specific vaccine. A detailed description of each measure is presented in section *Variable construction*. All trust variables were standardized so that effect sizes can be compared across trust measures and with respect to other studies. Figure 2 depicts the estimated effect sizes and the related confidence intervals (at 90% and 95% levels).

Our results suggested that individuals’ level of trust in the government, in pharmaceutical firms, and society are strong predictors of vaccine hesitancy. These coefficients were negative and statistically significant at the 95% confidence level. This means that persons with lower levels of trust in the national government, pharmaceutical companies, and society were more likely to be vaccine-hesitant. The estimated coefficient on governmental trust was about twice as large as the one for societal trust and trust in pharmaceutical firms. A one standard deviation higher level of trust in the government reduced the likelihood to be vaccine-hesitant by around five percentage points. Levels of trust in the national media and science appeared unrelated to vaccine hesitancy.

Regarding other variables included in the regression framework, we found that being female and vaccination status were negatively correlated with vaccine hesitancy (statistically significant at the 90% level). Prior literature on gender differences in vaccine hesitancy is rare for the context of Africa. Mainly focusing on COVID-19 it shows mixed findings^47–49^. Though gender differences are important to study, this topic is beyond the scope of this paper. Other individual-level controls seemed to rather be unrelated to vaccine hesitancy. The full set of regression results is shown in Supplementary Table B5 in Appendix B.

We performed several robustness checks to assess the sensitivity of our results. First, we re-estimated our main specification by using non-linear probit models (see Supplementary Fig. B1 in Appendix A). In addition to trust in government, society, and pharmaceutical companies, the coefficient of trust in science was negative and became statistically significant (90% level). However, this effect could not be confirmed in the sub-samples, though admittedly these samples had less statistical power (see Supplementary Figs. A2 and A3 in Appendix A). Second, we used an alternative dependent variable. More specifically, we replaced the previous measure of vaccine hesitancy with a variable that captured the confidence in vaccinations. The respective results (Supplementary Fig. A4 in Appendix A) were similar to our previous findings, demonstrating that trust in the government, society, and pharmaceutical firms were positively associated with vaccination confidence. Third, we used alternative definitions of our principal independent variables. Using regression specifications with binary trust measures that distinguished between persons with higher and lower trust we obtained similar results as before (see Supplementary Table B6 in Appendix B). Lastly, Supplementary Table B7 in Appendix B contains results from a range of further robustness checks related to (i) the clustering of standard errors and (ii) alternative covariate specifications. Columns (1) to (3) report estimates with standard errors being clustered at the country, vaccine-type, or country-vaccine-type level. Column (4) includes additional controls that measure respondents’ tendency to answer survey questions strategically (social desirability bias), while column (5) controls for treatment indicators of an experiment that was part of the survey for another study and unrelated to our topic. By and large, we found that the results related to trust in the government and society were robust to these model modifications.

### Context-specific effects

**Figure 3:**
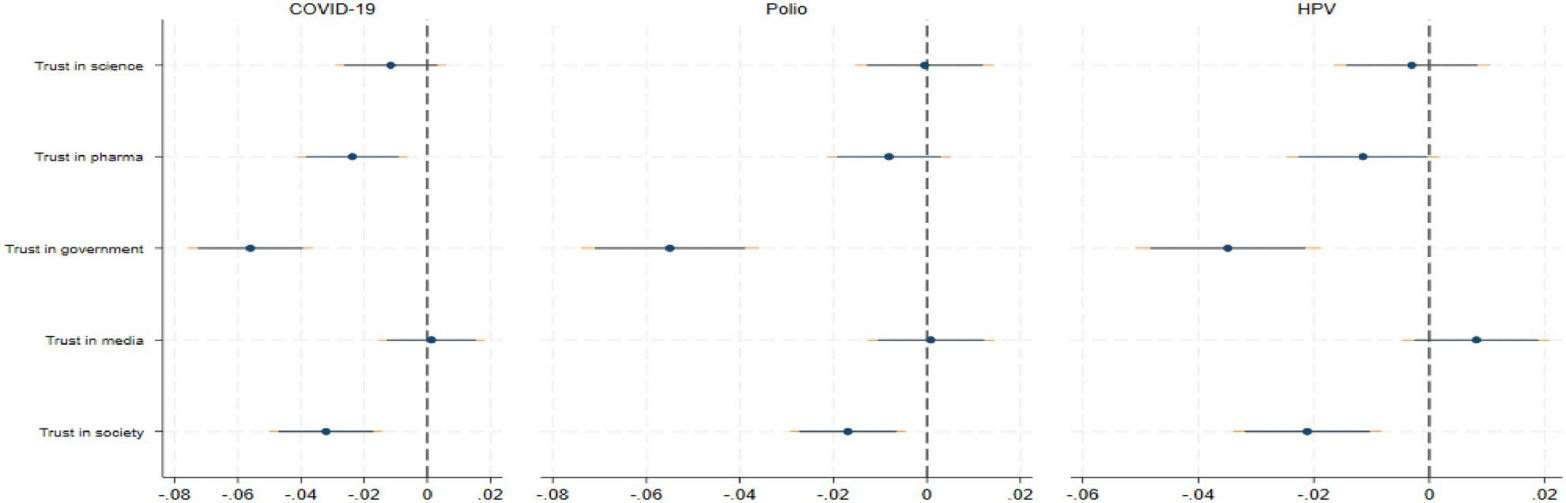
Trust and vaccine hesitancy by disease. The Figure reports coefficient estimates and confidence intervals at the 90 and 95 percent level. Results are obtained by OLS (see section “Empirical strategy”) for sub-samples. Controls included are age, gender, marital and employment status, wealth, education, religion, personality traits, and vaccination history as well as country and vaccine-type fixed effects. Robust standard errors are used.

We now turn to the analysis of context-specific effects. To investigate variations across study contexts, we re-estimated our main OLS specifications for different sub-samples. First, we focused on vaccine-type specific effects. As respondents were asked to state vaccination intentions on one of three vaccines only (randomized), each sub-sample consisted of about one third of the total number of observations. Figure 3 displays our results.

Overall, we found very consistent patterns. Across all three samples, trust in the government, pharmaceutical industry, and society was negatively correlated (conditionally) with vaccine hesitancy.

**Figure 4:**
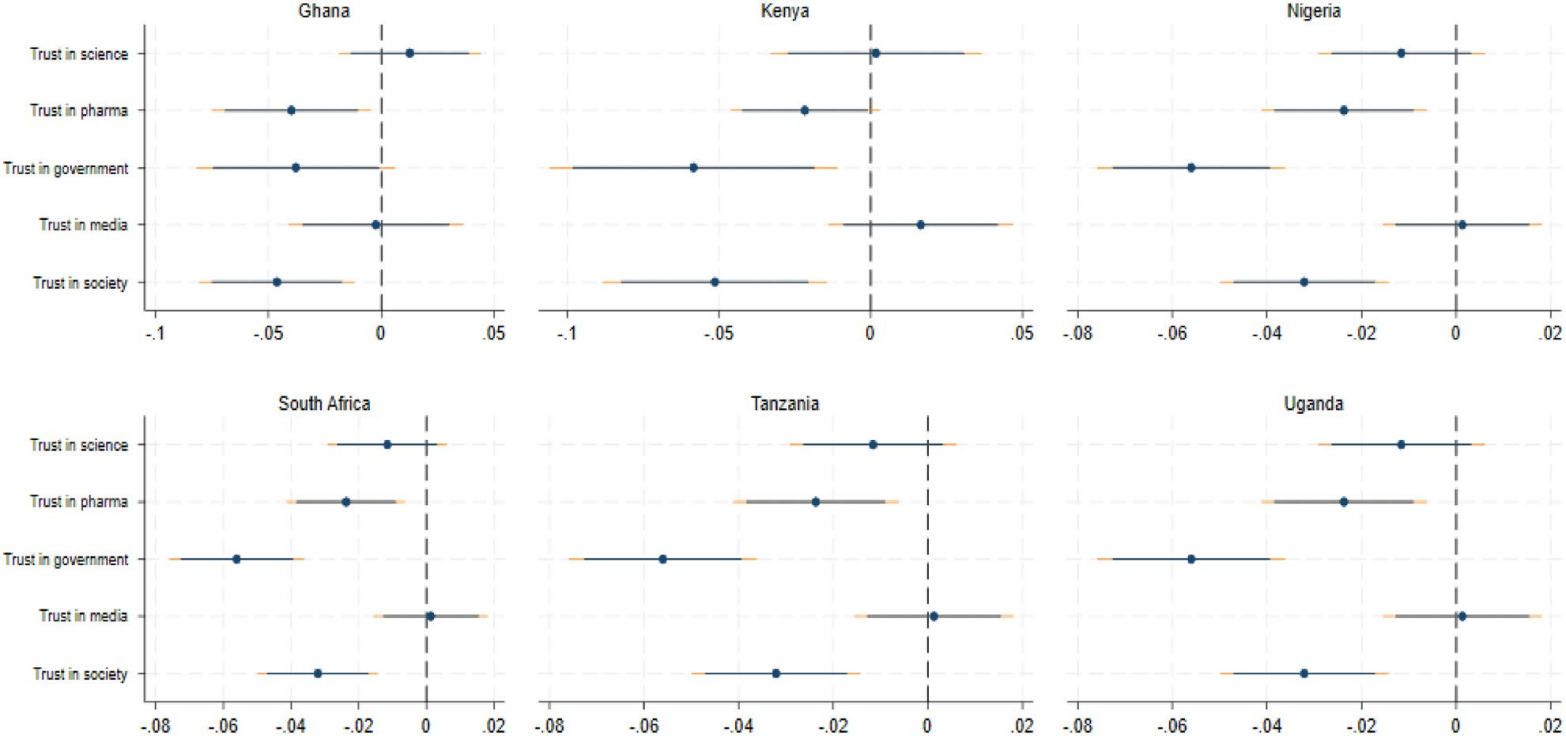
Trust and vaccine hesitancy by country. The Figure reports coefficient estimates and confidence intervals at the 90 and 95 percent level. Results are obtained by OLS (see section “Empirical strategy”) for sub-samples. Controls included are age, gender, marital and employment status, wealth, education, religion, personality traits, and vaccination history as well as country and vaccine-type fixed effects. Robust standard errors are used.

Second, we analysed country differences. Figure 4 presents the regression estimations for each of the six countries: Ghana, Kenya, Nigeria, South Africa, Tanzania, and Uganda.

For all countries, we found a statistically significant negative correlation between trust in the society with vaccine hesitancy. Trust in the government and in pharmaceutical firms was statistically significant and negatively correlated with vaccine hesitancy in the majority of countries. Exceptions were South Africa for trust in pharmaceutical firms and Tanzania for trust in the government. Trust in science seemed to be uncorrelated to vaccine hesitancy in most countries except for South Africa.

While COVID-19 and HPV are viruses that were circulating in all countries, not all countries in our study sample had recently experienced a polio outbreak. Moreover, the polio vaccine nOPV2 is distinct from the other two types of vaccines as it is only used in emergencies and not part of the routine immunization programs. This might cause a bias of the estimates related to polio if survey participants found it hard to imagine the hypothetical case of a polio outbreak in their country. To see in how far our results are sensitive to such a situation, we distinguished between countries that experienced a polio outbreak in the last few years and others and present the results of the sub-samples in Supplementary Fig. A5 in Appendix A. In both samples, we found a negative correlation between trust in the government and vaccine hesitancy. Additionally, we found a negative correlation between trust in the society and vaccine hesitancy but only for the sample of countries that have experienced a polio outbreak.

## Discussion

Immunization is one of the most cost-effective health interventions to tackle vaccine-preventable diseases. Across the world vaccine hesitancy is widely acknowledged to be one of the largest threats to achieve the widespread adoption and roll-out of vaccinations. As such the observed increasing rates of vaccine hesitancy pose a tremendous challenge for global health and the targets set under the Sustainable Development Goal #3 on good health and well-being. A better understanding of the determinants of vaccine hesitancy is pivotal to design effective policies to boost immunization rates.

In this study, we examined the relationship between trust and vaccine hesitancy. Leveraging primary data from six SSA countries, our analysis showed that low levels of trust in the government go along with a higher likelihood of vaccine hesitancy. Our results also emphasized the role of societal trust and social norms in the decision to get vaccinated. Specifically, we found that individuals are more likely to get vaccinated if (they believed that) others do so. Lastly, we found empirical evidence for a lack of trust in pharmaceutical companies to influence vaccine hesitancy.

The second part of our analysis relates to vaccine and country-specific effects in the relationship between trust and vaccine hesitancy. We showed that the relationship between trust and vaccine hesitancy was much less context-specific as expected. Most importantly, we demonstrated that the same trust factors that were responsible for vaccine hesitancy against COVID-19 helped explain vaccine hesitancy against other vaccines and diseases (HPV, polio). Moreover, the trust-related drivers of vaccine hesitancy were highly comparable across all six study countries.

Our results corroborate previous literature documenting that low levels of trust in the government is a pivotal predictor of vaccine hesitancy. We add to the existing literature by expanding the analysis of the trust-vaccine hesitancy nexus to other disease and vaccine contexts. Hence, a more comprehensive understanding of vaccine hesitancy in SSA has become possible. In contrast to several other studies, we found no robust evidence for a relationship between trust in media and science on vaccine hesitancy. Moreover, we provided new evidence on how trust in society in regards to social norms influences vaccine hesitancy. While our measure of societal trust is adjusted for the specific disease and vaccine type, previous research had to rely on very aggregated measures of societal trust (e.g. overall trust in strangers^12^). The inclusion of trust in society specified as social norm could be an explanation for the null findings of trust in media and science, if those factors are mainly operating through the societal channel.

Our study focused on six English-speaking African countries. While these countries comprise a diverse geographical, cultural, and health setting, it remains unclear in how far the relation between trust in crucial institutions and vaccine hesitancy applies to other African countries. Studies that were conducted in the context of COVID-19 in other African countries showed similar results, supporting the context-unspecific relation between trust in pivotal institutions and vaccine hesitancy. For instance, a negative relation between trust in pharmaceutical firms and vaccine hesitancy was found in Cameroon^50^. Several studies found a negative relation between vaccine hesitancy and trust in public institutions in the context of various African countries^8,51^.

A final thought relates to gender aspects. Preventive health behavior and preferences often tend to differ between men and women^52,53^. Therefore and considering that HPV vaccines are targeted towards young girls and women, trust-related effects on vaccine hesitancy might differ by gender. Split sample estimates that are illustrated in Supplementary Fig. A6 in Appendix A, however, revealed high similarities in the relationship between trust and vaccine hesitancy across gender and vaccine types.

Several policy conclusions can be derived on basis of our findings. First, our results imply a rather universal relationship between trust in the government and vaccine hesitancy, enabling the use of more targeted policies to address this specific trust issue. Second, to increase vaccination acceptance, policymakers should focus on emphasizing the social acceptance of vaccination. Third, when new vaccines enter the market, raised mistrust in pharmaceutical firms can decrease vaccination up-take.

Finally, we would like to point out some limitations of our study. First, the cross-sectional nature of data did not allow us to identify causal effects. By controlling for a large set of individual characteristics in combination with country and vaccine-type fixed effects, we, however, were able to control for several potentially confounding factors. Second, our measure of vaccine hesitancy captured respondents’ intention to get vaccinated. Bussink-Coorend et al.^54^ discussed the challenge to conceptualize vaccine hesitancy and the lack of a precise definition. While we show the robustness of our results to an alternative measure (vaccination confidence), we did not consider cognitive and affective aspects of indecisiveness. Third, using social media to recruit respondents, our sample was rather representative of a social media user population, but however, not representative of the national population in the respective countries. Lastly, participants in our survey were adults, yet polio and HPV vaccinations are mainly given to children and teenagers. While the prior literature showed that parents often decide for their children^55–57^, adult responses might be biased and not fully capture vaccine hesitancy of the target group.

## Methods

### Data collection and sample description

Our analyses were based on primary data that we collected between February to March 2023 via online surveys in six SSA countries (Ghana, Kenya, Nigeria, South Africa, Tanzania, and Uganda). The surveys were implemented on the UniPark platform. We recruited participants through paid advertisements on Facebook. Advertisements targeted all Facebook users in the target countries. The Facebook ads stated that survey participants had the chance to win phone credit upon successful completion of the survey. 12,975,764 persons saw the advertisements. Facebook users that were interested in participating could click on a link and were directed to our surveys on UniPark. Respondents had to be adults (18 years or older). Informed consent was obtained from all participants at the beginning of each survey. Respondents did not receive any compensation for survey participation. However, in order to motivate participation, we distributed twelve 5G mobile phone credits among all survey participants. The twelve persons were randomly selected after the finalization of the surveys. We obtained ethical approval from the ethical commission of the medical association in Hamburg, called ‘Ethik-Kommission der Ar̈ztekammer Hamburg’. All methods were performed in accordance with relevant guidelines and regulations.

The surveys gathered information on a number of individual characteristics, attitudes towards health topics, health knowledge, and health behavior. The surveys were completed by 5203 participants. During the data cleaning process, we dropped observations that did not pass quality controls (attention checks; participants that typically live outside of the targeted countries). The majority of participants were from Kenya. The average age of respondents was 29.15 years. 35% of respondents were female. Summary statistics on the main variables are presented in Supplementary Table B2 in Appendix B. In general, the sample appeared to be representative of the social media user population. It was, however, not representative of the general population.

### Experimental set-up

The survey comprised three parts. Part 1 collected information on the socio-demographic characteristics of the respondents, including age, gender, religion, wealth, among others. In Part 2 respondents were randomly assigned to one of the following three vaccine-type groups: COVID-19, HPV, polio. Respondents in the COVID-19 group received a short text about the COMIRNATY BA.4/BA.5 vaccine, whereas respondents in the HPV group received a short text describing the GARDASIL4 and CERVARIX vaccine. Respondents in the polio group read a text about the nOPV2 vaccine. The texts are presented in Supplementary Appendix C together with the survey questionnaire. In Part 3 all respondents received identical survey questions measuring trust into the different institutions and vaccine hesitancy. We performed balance tests that are presented in Supplementary Table B9 in Appendix B. We showed that overall the samples are highly comparable with respect to almost all socio-demographic characteristics. An exception was gender that we included as control in all regressions.

### The three vaccines

We considered information regarding three vaccines in the survey: the Comirnaty Ba.4/Ba.5 vaccine against COVID-19, the Gardasil-4 against HPV, and the nOPV2 vaccine against polio. We selected these three vaccines because of their current relevance. For most countries these vaccines have been recently integrated in the national immunization program or are planned to do so. LMICs started HPV vaccination for young girls in 2012. Yet, the use of the GARDASIL4 and CERVARIX vaccine is currently small. Nigeria and Ghana planned to include the vaccine in their vaccination program this year. Kenya, Tanzania, Uganda, and South Africa have included it in their national campaigns already. Referring to COVID-19, the COMIRNATY BA.4/BA.5 vaccine is an adapted version of the mRNA COVID-19 vaccine Comirnaty (Pfizer/BioNTech) and recommended for use in people age 12 and older. It is approved in several countries (EU, US, Canada), but still in the process to be approved in most African countries. Regarding polio, the nOPV2 vaccine is a novel oral poliomyelitis (polio) vaccine that was developed to address the increasing risk of vaccine-derived polio-virus type 2 (cVDPV2). It received a recommendation for use in November 2020. The vaccine is used only for polio outbreak response. Hence, it is distributed according to outbreaks. It has been used in several African countries. The highest share of nOPV2 doses went to Nigeria^58^. All three vaccines have been approved by recognized authorities. For instance, the COVID-19 and HPV vaccine received EMA approval in 2006 and 2022^59,60^, and the polio vaccine has been authorized for the WHO Emergency Use Listing^61^.

### Variable construction

Our outcome of interest was vaccine hesitancy. In the survey, respondents were presented a short text about a new vaccine and asked about their agreement with the following statement “I intend to get vaccinated against [vaccine type] with the new vaccine or will encourage one of my family members to do so”. The question varies across randomized sub-groups (COVID-19, polio, HPV). Respondents could answer on a 7-point Likert scale that ranged from 0 “strongly disagree” to 6 “strongly agree”. Based on this survey item, we constructed a binary indicator of vaccine hesitancy that takes the value one if responses to the survey item were below 3. For robustness checks, we used vaccination confidence as an alternative outcome of interest. Vaccination confidence was an index (average value) constructed on basis of three survey items: (i) “I worry about the side effects of vaccines.”, (ii) “After getting vaccinated, I feel protected.” and (iii) “I believe that vaccines often cause more harm than good.” Respondents gave their agreement to the three statements on a 7 point Likert scale. The first and third item entered the score in reversed form.

The main explanatory variables in our study were measures of trust in various institutions. We standardized all trust variables to be able to compare magnitudes. Trust in science and media was measured with the respondents’ evaluation of the trustworthiness of health-related information from the named institution on a 4 point Likert scale ranging from 0 “not at all trustworthy” to 3 “a lot trustworthy”. Trust in pharmaceutical firms was measured with the respondents agreement to the following question item “I believe that Western countries use pharmaceutical companies to exploit African people for their own purposes.” in a reverse order. Participants could agree or disagree on a 7 point Likert scale. We constructed an index for trust in government using the average score of the following three question items: (i) “In your opinion, how trustworthy are health-related information from the government?”, answered on a 4 point Likert scale, (ii) “How much do you trust in the ministry of health in your country?” answered with a 7 point Likert scale, and (iii) the respondent’s agreement to the statement “I believe that governmental regulations in my country ensure quality vaccines and drugs.” on a 7 point Likert scale. Lastly, trust in society was assessed with the respondent’s belief about the share of persons in the country that would like to get vaccinated with the described vaccine. Supplementary Table B8 in Appendix B presents the correlation matrix of our trust measures.

Lastly, we considered a wide range of individual characteristics. First, the following socio-demographic characteristics were considered: gender (0 = male, 1 = female), age in number of years, highest level of education (ranging from no education to university/tertiary education), married (indicator whether person is married), work status ((self-)employed = 1, 0 otherwise), wealth (very poor, poor, average, rich, very rich relative to others), and religion (Islam, Christianity, traditional beliefs, no religion, other). The socio-economic factors were based on self-reported statements. Moreover, we assessed two personality traits (agreeableness and openness to new experiences) using TIPI personality test items. Moreover, we controlled for past vaccinations including indicator variables that identify persons that have been vaccinated against polio, HPV, and COVID-19 based on self-reported responses.

### Empirical strategy

In order to estimate the relationship between trust in different institutions and vaccine hesitancy, we run a multivariate linear probability model. We regressed an indicator variable that captures whether an individual intends to get vaccinated on trust measures using the following OLS model:

1 $$\begin{aligned} Y_{ivc} = \textbf{Trust}'_{ic}\beta + \textbf{X}'_{ic}\gamma + \eta _{v} + \phi _{c} + \varepsilon _{ivc}, \end{aligned}$$

where $Y_{ivc}$ was a binary indicator that captures whether respondent *i* in country *c* had no intention to get vaccinated with vaccine *v*. The principal explanatory variables of institutional trust were included in the matrix $\textbf{Trust}_{ic}$. We considered trust in science, the national government, national media, pharmaceutical firms, and society. $\textbf{X}_{ic}$ was a matrix of individual-level characteristics including socio-demographics (age, gender, religion, education, wealth, employment and marital status, and personality traits) and a person’s own vaccination history regarding polio, HPV, and COVID-19. $\eta _{v}$ related to vaccine-type fixed effects, $\phi _{c}$ represented country fixed effects, and $\varepsilon _{iv}$ was the error term. Standard errors were robust. As common with cross-sectional regressions, the specification estimated conditional correlations between trust and vaccine hesitancy. We do not claim any causality as we cannot completely rule out omitted variable bias or reverse causality. All statistical analyses were conducted with Stata 18.

### Supplementary Information

Supplementary Information.

## Data Availability

Data and code of the analysis is available upon request from the corresponding author.
