# Supplementary material for: Vaccine hesitancy and trust in sub-Saharan Africa
Source: Sci Rep. 2024 May 13;14:10860. doi: 10.1038/s41598-024-61205-0 (PMC11091197; doi:10.1038/s41598-024-61205-0)
Supplement: Supplementary file 1 — Supplementary Information. [file 41598_2024_61205_MOESM1_ESM.pdf]

Appendix

A Figures

Figure A1. Robustness check: Probit model of the relation between trust and vaccine hesitancy

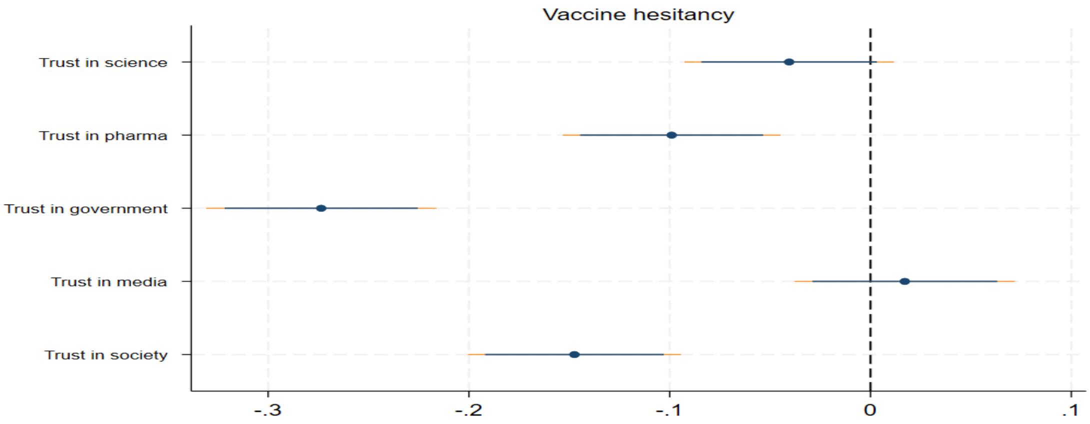

Note: The figure reports coefficient estimates and confidence intervals at the 90 and 95 percent level of the probit regression model that is described in section *Empirical strategy*. Controls included are age, gender, marital and employment status, wealth, education, religion, personality traits, and vaccination history as well as country and vaccine-type fixed effects. Standard errors are robust.

Figure A2. Robustness check: Probit model of the relation between trust and vaccine hesitancy by vaccine type

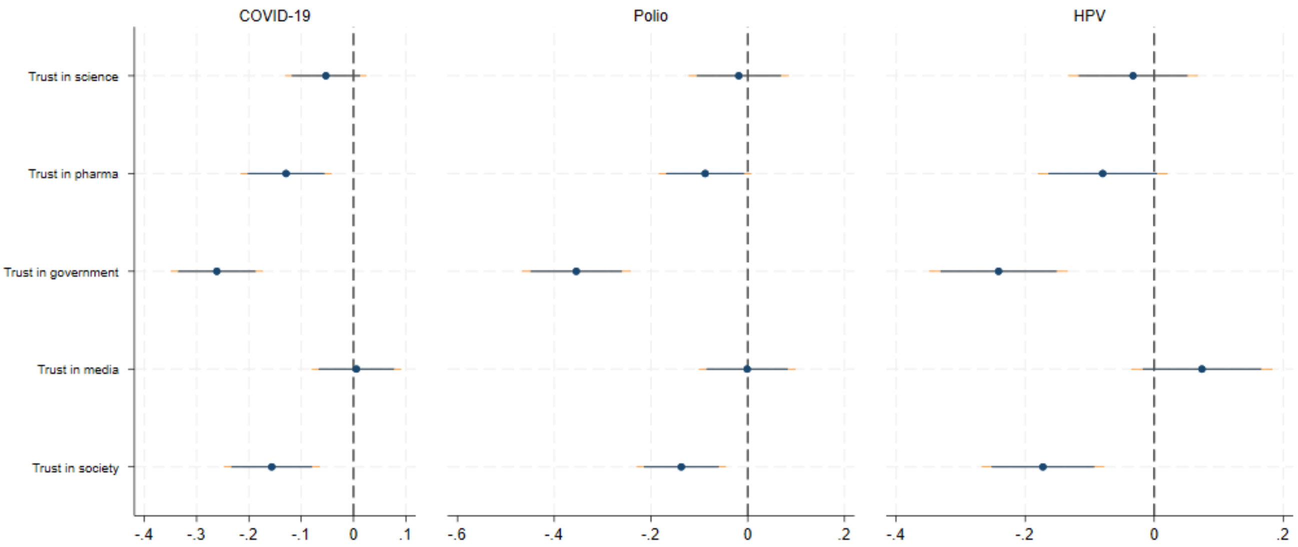

Note: The figure reports coefficient estimates and confidence intervals at the 90 and 95 percent level of the probit regression model that is described in section *Empirical strategy* of sub-samples by vaccine type. Controls included are age, gender, marital and employment status, wealth, education, religion, personality traits, and vaccination history as well as country and vaccine-type fixed effects. Standard errors are robust.

Figure A3. Robustness check: Probit model of the relation between trust and vaccine hesitancy by country

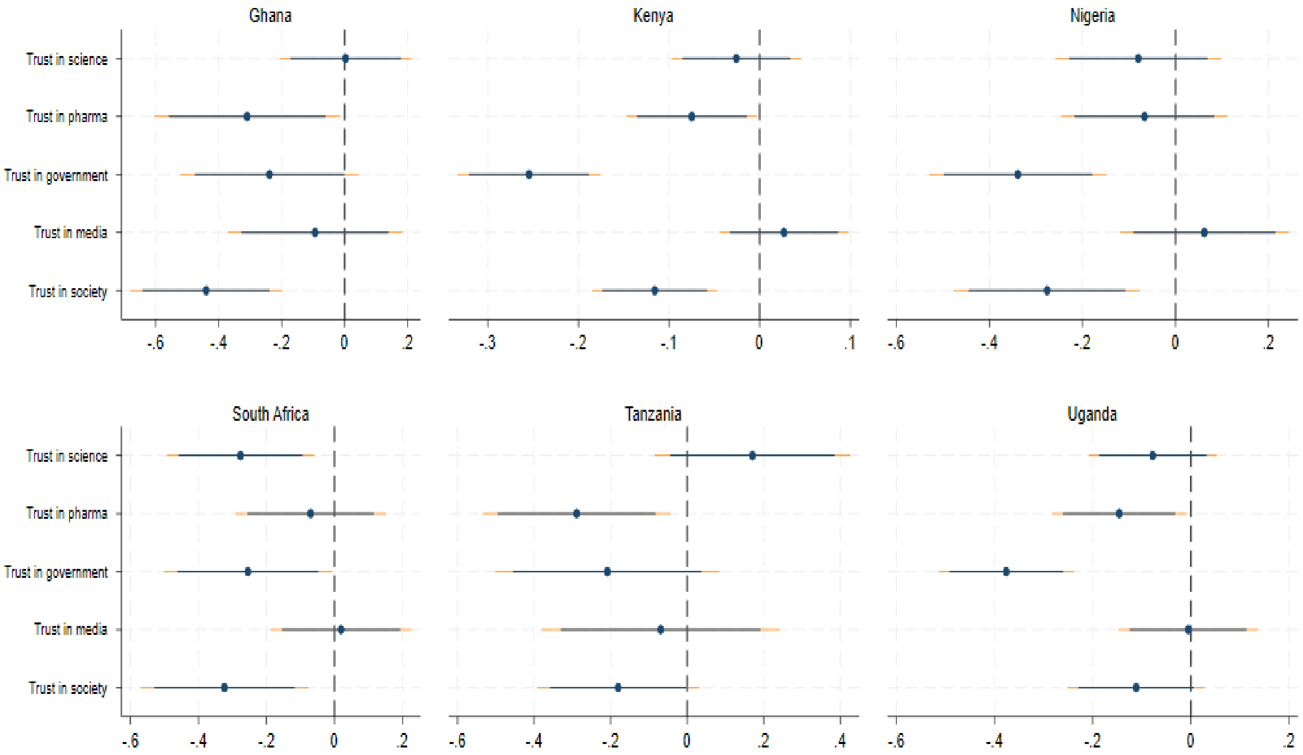

Note: The figure reports coefficient estimates and confidence intervals at the 90 and 95 percent level of the probit regression model that is described in section *Empirical strategy* in six sub-samples divided by country. Controls included are age, gender, marital and employment status, wealth, education, religion, personality traits, and vaccination history as well as country and vaccine-type fixed effects. Standard errors are robust.

Figure A4. Robustness check: Trust and vaccination confidence

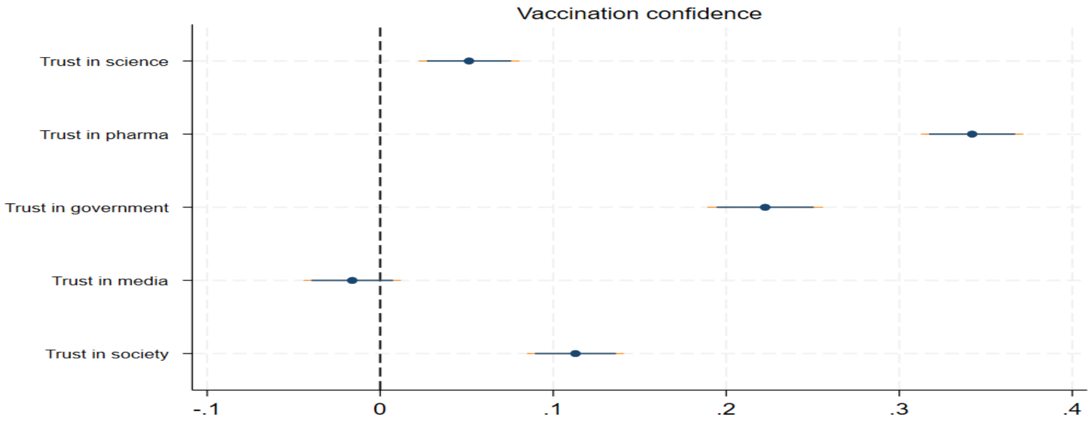

Note: The figure reports coefficient estimates and confidence intervals at the 90 and 95 percent level of the OLS regression model that is described in section *Empirical strategy*. Dependent variable is an index of vaccine confidence as defined in section **??** Vaccine confidence is regressed on various trust measures and individual controls. Controls included are age, gender, marital and employment status, wealth, education, religion, personality traits, and vaccination history as well as country and vaccine-type fixed effects. Standard errors are robust.

Figure A5. Heterogeneous effects by recent polio outbreaks

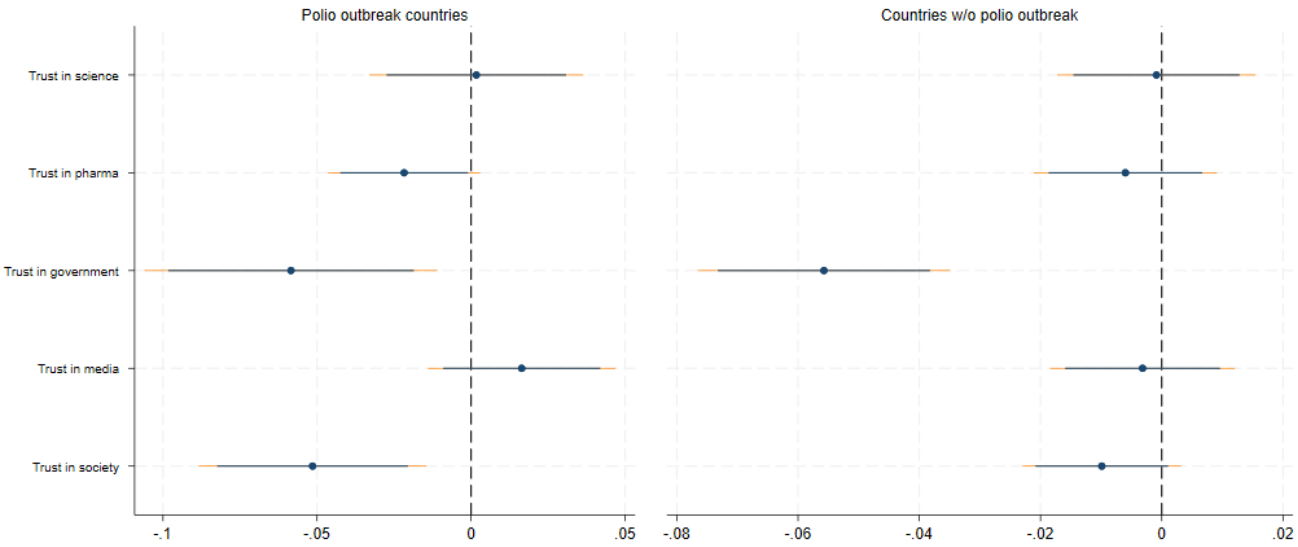

Note: The figure reports coefficient estimates and confidence intervals at the 90 and 95 percent level of the OLS regression model that is described in section *Empirical strategy* for countries that had a recent polio outbreak (Ghana and Nigeria) and others for the polio sub-sample. Controls included are age, gender, marital and employment status, wealth, education, religion, personality traits, and vaccination history as well as country and vaccine-type fixed effects. Standard errors are robust.

Figure A6. Gender effects: Trust and vaccination hesitancy

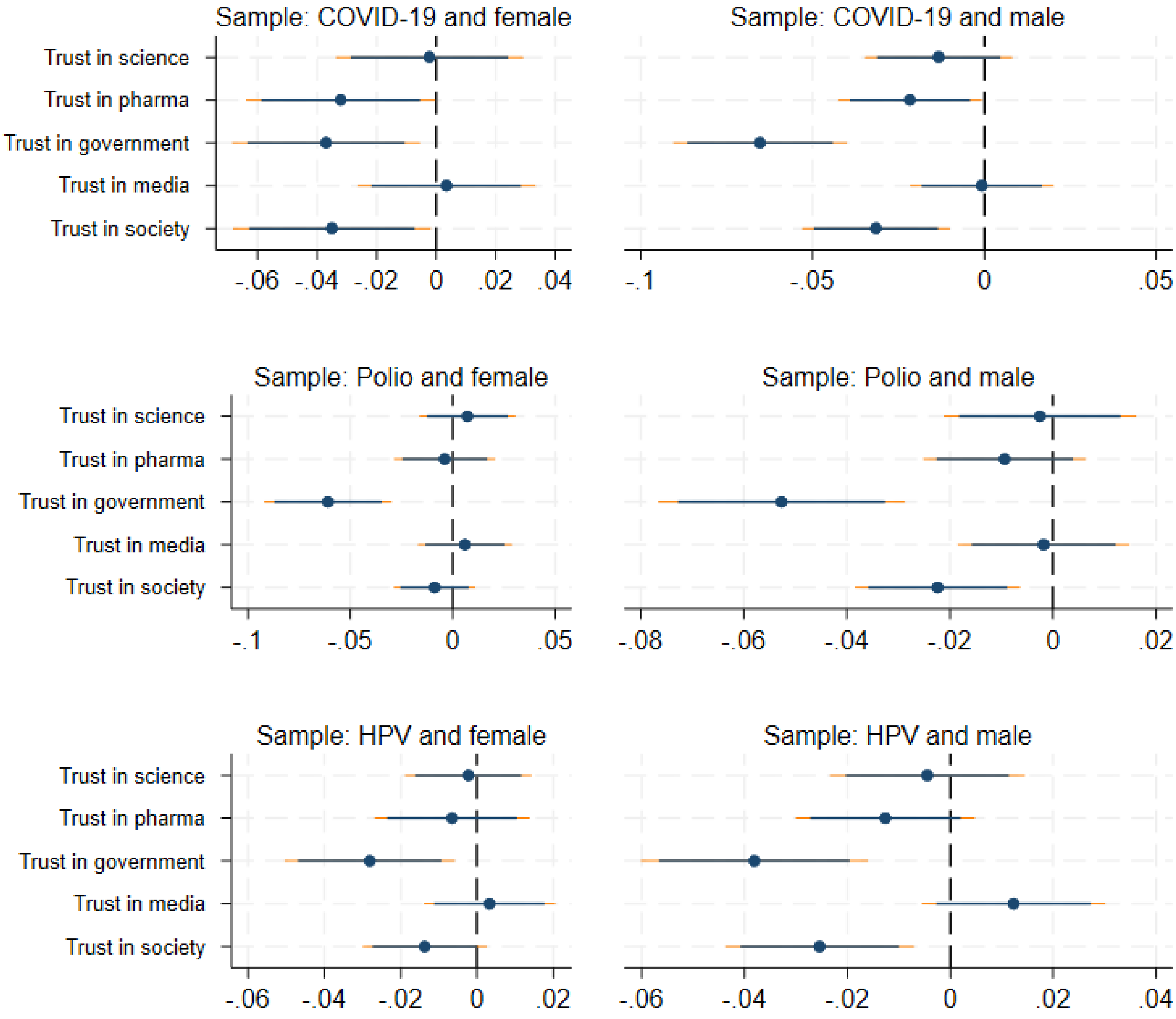

Note: The figure reports coefficient estimates and confidence intervals at the 90 and 95 percent level of the OLS regression model that is described in section *Empirical strategy* for male and female separately as well as by vaccine type. Controls included are age, gender, marital and employment status, wealth, education, religion, personality traits, and vaccination history as well as country and vaccine-type fixed effects. Standard errors are robust.

B Tables

Table B1. Observations by country

Country	Observations
Ghana	297
Kenya	3,062
Nigeria	611
South Africa	239
Tanzania	198
Uganda	796
Total	5,203

Table B2. Summary statistics

	Mean	SD	Min.	Max.	Obs.
Vaccine hesitancy	0.09	0.29	0.00	1	5203
Trust in science	-0.00	1.00	-3.36	1	5203
Trust in pharmaceutical firms	0.00	1.00	-1.89	1	5203
Trust in government	0.00	1.00	-3.65	2	5203
Trust in media	0.00	1.00	-2.53	1	5203
Trust in society	-0.00	1.00	-2.73	2	5203
Age (no. of years)	29.15	8.78	18.00	75	5203
Female	0.35	0.48	0.00	1	5203
Married	0.32	0.47	0.00	1	5203
(Self-)employed	0.56	0.50	0.00	1	5203
Wealth	1.90	0.60	0.00	4	5203
No or primary education	0.02	0.14	0.00	1	5203
Secondary education	0.24	0.43	0.00	1	5203
Christian	0.87	0.33	0.00	1	5203
Muslim	0.09	0.29	0.00	1	5203
Other religion	0.04	0.19	0.00	1	5203
Tertiary education	0.73	0.44	0.00	1	5203
Agreeableness	2.14	2.30	-6.00	6	5203
Openness	1.16	2.02	-6.00	6	5203

Table B3. Summary statistics by country

	Mean	SD	Min.	Max.	Obs.
			Ghana		
Vaccine hesitancy	0.09	0.29	0.00	1	297
Trust in science	-0.02	1.02	-3.36	1	297
Trust in pharmaceutical firms	-0.27	0.96	-1.89	1	297
Trust in government	-0.22	0.95	-3.33	2	297
Trust in media	-0.14	1.00	-2.53	1	297
Trust in society	0.06	0.94	-2.73	2	297
Age (no. of years)	29.99	8.46	18.00	75	297
Female	0.37	0.48	0.00	1	297
Married	0.36	0.48	0.00	1	297
(Self-)employed	0.64	0.48	0.00	1	297
Wealth	1.99	0.56	0.00	4	297
No or primary education	0.02	0.14	0.00	1	297
Secondary education	0.24	0.43	0.00	1	297
Christian	0.79	0.41	0.00	1	297
Muslim	0.17	0.37	0.00	1	297
Other religion	0.04	0.20	0.00	1	297
Tertiary education	0.74	0.44	0.00	1	297
Agreeableness	2.25	2.29	-6.00	6	297
Openness	1.12	1.82	-5.00	6	297
			Kenya		
Vaccine hesitancy	0.08	0.28	0.00	1	3062
Trust in science	0.03	0.98	-3.36	1	3062
Trust in pharmaceutical firms	0.10	0.99	-1.89	1	3062
Trust in government	0.13	0.93	-3.33	2	3062
Trust in media	0.03	0.98	-2.53	1	3062
Trust in society	0.00	1.00	-2.73	2	3062
Age (no. of years)	28.92	8.15	18.00	75	3062
Female	0.34	0.48	0.00	1	3062
Married	0.36	0.48	0.00	1	3062
(Self-)employed	0.55	0.50	0.00	1	3062
Wealth	1.89	0.58	0.00	4	3062
No or primary education	0.02	0.14	0.00	1	3062
Secondary education	0.21	0.41	0.00	1	3062
Christian	0.94	0.24	0.00	1	3062
Muslim	0.03	0.17	0.00	1	3062
Other religion	0.03	0.18	0.00	1	3062
Tertiary education	0.77	0.42	0.00	1	3062
Agreeableness	2.27	2.31	-6.00	6	3062
Openness	1.22	2.07	-6.00	6	3062
			Nigeria		
Vaccine hesitancy	0.08	0.28	0.00	1	611
Trust in science	0.05	1.00	-3.36	1	611
Trust in pharmaceutical firms	-0.08	0.92	-1.89	1	611
Trust in government	-0.35	0.98	-3.65	2	611
Trust in media	-0.01	1.01	-2.53	1	611
Trust in society	-0.01	0.93	-2.73	2	611
Age (no. of years)	27.73	8.63	18.00	67	611
Female	0.40	0.49	0.00	1	611

Married	0.29	0.45	0.00	1	611
(Self-)employed	0.56	0.50	0.00	1	611
Wealth	1.94	0.58	0.00	4	611
No or primary education	0.01	0.12	0.00	1	611
Secondary education	0.21	0.41	0.00	1	611
Christian	0.67	0.47	0.00	1	611
Muslim	0.31	0.46	0.00	1	611
Other religion	0.02	0.13	0.00	1	611
Tertiary education	0.78	0.42	0.00	1	611
Agreeableness	2.33	2.21	-6.00	6	611
Openness	1.17	1.91	-6.00	6	611
			South Africa		
Vaccine hesitancy	0.17	0.38	0.00	1	239
Trust in science	-0.12	1.05	-3.36	1	239
Trust in pharmaceutical firms	-0.40	1.05	-1.89	1	239
Trust in government	-0.42	1.14	-3.33	2	239
Trust in media	0.02	1.10	-2.53	1	239
Trust in society	-0.04	1.01	-2.55	2	239
Age (no. of years)	36.61	14.47	18.00	75	239
Female	0.66	0.48	0.00	1	239
Married	0.21	0.41	0.00	1	239
(Self-)employed	0.52	0.50	0.00	1	239
Wealth	1.82	0.55	0.00	4	239
No or primary education	0.03	0.18	0.00	1	239
Secondary education	0.45	0.50	0.00	1	239
Christian	0.82	0.38	0.00	1	239
Muslim	0.03	0.17	0.00	1	239
Other religion	0.15	0.35	0.00	1	239
Tertiary education	0.51	0.50	0.00	1	239
Agreeableness	1.79	2.31	-6.00	6	239
Openness	1.22	1.91	-5.00	6	239
			Tanzania		
Vaccine hesitancy	0.11	0.32	0.00	1	198
Trust in science	0.01	1.05	-3.36	1	198
Trust in pharmaceutical firms	-0.04	1.06	-1.89	1	198
Trust in government	0.24	0.91	-2.36	2	198
Trust in media	0.09	1.00	-2.53	1	198
Trust in society	0.02	1.18	-2.73	2	198
Age (no. of years)	28.78	8.88	18.00	66	198
Female	0.17	0.38	0.00	1	198
Married	0.27	0.45	0.00	1	198
(Self-)employed	0.55	0.50	0.00	1	198
Wealth	2.05	0.71	0.00	4	198
No or primary education	0.02	0.14	0.00	1	198
Secondary education	0.32	0.47	0.00	1	198
Christian	0.79	0.41	0.00	1	198
Muslim	0.16	0.37	0.00	1	198
Other religion	0.05	0.22	0.00	1	198
Tertiary education	0.66	0.47	0.00	1	198
Agreeableness	1.55	2.21	-6.00	6	198
Openness	0.91	1.86	-5.00	6	198
			Uganda		
Vaccine hesitancy	0.10	0.30	0.00	1	796

Trust in science	-0.10	1.04	-3.36	1	796
Trust in pharmaceutical firms	-0.09	1.01	-1.89	1	796
Trust in government	-0.10	1.12	-3.33	2	796
Trust in media	-0.09	1.05	-2.53	1	796
Trust in society	-0.02	1.02	-2.73	2	796
Age (no. of years)	28.70	8.01	18.00	70	796
Female	0.31	0.46	0.00	1	796
Married	0.25	0.43	0.00	1	796
(Self-)employed	0.60	0.49	0.00	1	796
Wealth	1.86	0.71	0.00	4	796
No or primary education	0.02	0.14	0.00	1	796
Secondary education	0.32	0.47	0.00	1	796
Christian	0.83	0.37	0.00	1	796
Muslim	0.12	0.32	0.00	1	796
Other religion	0.05	0.22	0.00	1	796
Tertiary education	0.66	0.47	0.00	1	796
Agreeableness	1.72	2.28	-5.00	6	796
Openness	1.00	2.04	-6.00	6	796

Table B4. Statistics on national vaccination rates

Country	HPV	COVID-19	Polio
Ghana	-	57.6%	98%
Kenya	29%	47.8%	91%
Nigeria	-	69.1%	56%
South Africa	79%	47.9%	86%
Tanzania	70%	100%	81%
Uganda	99%	57.6%	90%

Note: The table reports vaccination rates per country. HPV vaccination rates are estimates provided by the UNICEF Immunization data portal and gives the percentage of teenagers turning 15 in 2022 that have received any HPV vaccine. No information is available for Ghana and Nigeria. COVID-19 vaccination rates give the percentage of persons that received any COVID-19 vaccine of the full vaccine-eligible population based on information from Africa CDC website (Official Regional Collaborating Centre Statistics and Member State Reports) in most recent years. Polio vaccination rates refer to Polio immunization (3 doses) rates among one-year olds from WHO and UNICEF in the 2021 cohort.

Table B5. Main regression specification

	Vaccine hesitancy
Trust in science	-0.006
	(0.005)
Trust in pharmaceutical firms	-0.014***
	(0.004)
Trust in government	-0.048***
	(0.005)
Trust in media	0.002
	(0.004)
Trust in society	-0.023***
	(0.004)
Age (no. of years)	0.000
	(0.001)
Female	-0.020**
	(0.008)
Married	-0.001
	(0.009)
(Self-)employed	0.003
	(0.008)
Wealth	0.002
	(0.006)
No or primary education	0.004
	(0.028)
Secondary education	-0.006
	(0.009)
Christian	-0.038
	(0.026)
Muslim	-0.025
	(0.029)
Other religion	0.000
	(.)
Agreeableness	0.001
	(0.002)
Openness	-0.002
	(0.002)
Vaccinated HPV	-0.001
	(0.012)
Vaccinated polio	-0.034**
	(0.016)
Vaccinated COVID-19	-0.037***
	(0.010)
Observations	5,203
R^2	0.076

Note: The table reports coefficient estimates and standard errors of the OLS regression of vaccine hesitancy on the trust measures and various individual characteristics as shown in the table. Standard errors are clustered at the level of vaccination type. Regressions include vaccination type and country fixed effects. ***,*,* denote significance at 1, 5 and 10%. We tested for multicollinearity among the trust measures. The VIF is close to 1, reassuring us that collinearity does not confound the analysis.

Table B6. Robustness check: Binary measure of trust variables

	Vaccine hesitancy
Trust in science (binary)	-0.013
	(0.007)
Trust in pharma. firms (binary)	-0.024**
	(0.009)
Trust in government (binary)	-0.063***
	(0.007)
Trust in media (binary)	-0.011
	(0.006)
Trust in society (binary)	-0.046***
	(0.009)
Observations	5,203
R^2	0.059

Note: The table reports coefficient estimates and standard errors of the OLS regression of vaccine hesitancy on the trust measures and various individual characteristics. Controls included are age, gender, marital and employment status, wealth, education, religion, personality traits, and vaccination history as well as country and vaccine-type fixed effects. Standard errors are robust. Trust measures are binary measures that report above median trust in the respective institution. ***,*,* denote significance at 1, 5 and 10%.

Table B7. Robustness check: Modification of standard errors and further control variables

	Dep. var.: Vaccine hesitancy				
	Clustered standard errors			Further controls	
	(1)	(2)	(3)	(4)	(5)
	country	vaccine-type	country + vaccine-type	Social desirable index	Treatment effects
Trust in science	-0.006	-0.006	-0.006	-0.004	-0.006
	(0.004)	(0.003)	(0.004)	(0.007)	(0.005)
Trust in pharmaceutical firms	-0.014**	-0.014*	-0.014	-0.009	-0.014***
	(0.004)	(0.005)	(0.006)	(0.009)	(0.004)
Trust in government	-0.048***	-0.048**	-0.048**	-0.046***	-0.048***
	(0.005)	(0.007)	(0.007)	(0.011)	(0.005)
Trust in media	0.002	0.002	0.002	0.001	0.002
	(0.001)	(0.003)	(0.002)	(0.008)	(0.004)
Trust in society	-0.023**	-0.023**	-0.023*	-0.017**	-0.023***
	(0.006)	(0.004)	(0.006)	(0.007)	(0.004)
Social concern index				-0.004	
				(0.006)	
Treatment 1					0.002
					(0.009)
Treatment 2					0.004
					(0.009)
Observations	5,203	5,203	5,203	1,607	5,203
R^2	0.076	0.076	0.076	0.072	0.076

Note: The table reports coefficient estimates and standard errors of the OLS regression of vaccine hesitancy on the trust measures and various individual characteristics. Controls included are age, gender, marital and employment status, wealth, education, religion, personality traits, and vaccination history as well as country and vaccine-type fixed effects. Columns 1 and 2 change the level of clusters of the standard errors. In column 1 standard errors are clustered at the country level. In column 2 they are clustered at the level of country and vaccination type. Regressions of columns 3 and 4 include further control variables as shown in the table. Social desirability index measures the individual tendency to answer in a socially desirable form. Treatment 1 and 2 refers to treatment effects of an experiment that is included in the African Health Survey. Regressions include vaccination type and country fixed effects. ***,*,* denote significance at 1, 5 and 10%.

C Survey instruments

Survey questions

Section A: Demographics, personality, and social desirability

No.	Question	Answering options
To begin with, we have some questions about you and your life.		
A 01	How old are you?	____Years
A 02	What is your gender?	0 Female<br>1 Male
A 03	What country do you currently live in?	0 Nigeria<br>1 Ghana<br>2 Tanzania<br>3 Uganda<br>4 Kenya<br>5 South Africa<br>9 Other
A 04	What is your highest level of education (completed)?	0 No schooling / primary school<br>1 Junior secondary<br>2 Senior secondary<br>3 University/tertiary education<br>9 Other: ____________
A 05	Are you married?	0 Single<br>1 Living together as married<br>2 Married<br>3 Divorced/Widowed/Separated
A 06	What was your work status in the previous week?	0 Employed<br>1 Self-employed/Freelancer<br>2 Temporarily not working (e.g. holidays)<br>3 Unemployed<br>4 Other (e.g. Student, not looking for any work)
A 07	Compared to other people in your country, would you consider yourself to be …?	0 very poor<br>1 poor<br>2 average<br>3 rich<br>4 very rich
A 08	What is your religion?	0 Islam<br>1 Christianity<br>2 Traditional beliefs (e.g. voodoo)<br>3 No religion (agnostic, atheist)<br>4 Other (e.g. Buddhism, Hinduism, Taoism)<br>98 Prefer not to answer
	**Personality traits based on TIPI**	
A 09	Would you agree with the following statement? **"I see myself as someone who…."**<br><br>*Please state your agreement on a scale from 1 = Strongly disagree to 7 = Strongly agree.*	
A 09.1	…is generally trusting.	0 Strongly disagree<br>1 Disagree

		2 Somewhat disagree<br>3 Neither agree nor disagree<br>4 Somewhat agree<br>5 Agree<br>6 Strongly agree
A 09.2	…tends to find fault with others.	0 Strongly disagree<br>1 Disagree<br>2 Somewhat disagree<br>3 Neither agree nor disagree<br>4 Somewhat agree<br>5 Agree<br>6 Strongly agree
A 09.3	…has few artistic interests.	0 Strongly disagree<br>1 Disagree<br>2 Somewhat disagree<br>3 Neither agree nor disagree<br>4 Somewhat agree<br>5 Agree<br>6 Strongly agree
A 09.4	…has an active imagination.	0 Strongly disagree<br>1 Disagree<br>2 Somewhat disagree<br>3 Neither agree nor disagree<br>4 Somewhat agree<br>5 Agree<br>6 Strongly agree
	**Social concern index**	
A 10	On a scale from 1 to 5, where 1 means "it does not fit me" and 5 means "it fits me perfectly", comment on the following statement:<br><u>"It is important for me not to be considered selfish."</u>	0 It does not fit me at all<br>1 It rather does not fit me.<br>2 It neither fits me nor does not fit me.<br>3 It rather fits me.<br>4 It fits me perfectly.
A 11	On a scale from "1" (never) to "5" (often), comment on the following statements.	
A 11.1	I have worked for a charitable organization.	0 Never<br>1 One time before<br>2 Seldom<br>3 Sometimes<br>4 Often
A 11.2	I have donated blood.	0 Never<br>1 One time before<br>2 Seldom<br>3 Sometimes<br>4 Often

Section B: Trust

No.	Question	Code
B 01	In your opinion, how trustworthy are health-related information from the following sources?	

B 01.1	Science & Research	0 Not at all trustworthy<br>1 Just a little trustworthy<br>2 Somewhat trustworthy<br>3 A lot trustworthy
B 01.2	Traditional media (print, TV, radio)	0 Not at all trustworthy<br>1 Just a little trustworthy<br>2 Somewhat trustworthy<br>3 A lot trustworthy
B 01.3	Government	0 Not at all trustworthy<br>1 Just a little trustworthy<br>2 Somewhat trustworthy<br>3 A lot trustworthy
B 02	Next, we would like to ask you a few questions about your trust in different organizations. Please indicate for each how much you trust in it.	
B 02.1	The ministry of health in your county	0 No trust at all<br>1 Hardly trust<br>2 Just a little trust<br>3 Somewhat trust<br>4 Mostly trust<br>5 Trust a lot<br>6 Complete trust
B 03	In the following we want to know more about your attitudes towards vaccines. Please state how strongly you agree or disagree with the statement on a scale from 1 to 7.	
B 03.1	I believe that governmental regulations in my country ensure quality vaccines and drugs.	0 Strongly disagree<br>1 Disagree<br>2 Somewhat disagree<br>3 Neither agree nor disagree<br>4 Somewhat agree<br>5 Agree<br>6 Strongly agree
B 03.2	I believe that Western countries use pharmaceutical companies to exploit African people for their own purposes.	0 Strongly disagree<br>1 Disagree<br>2 Somewhat disagree<br>3 Neither agree nor disagree<br>4 Somewhat agree<br>5 Agree<br>6 Strongly agree
B 04	Imagine the national government rolls out a vaccine campaign for all adults (18+). What do you think is the share of adults who would want to get vaccinated?<br>*Please enter a number between 0 and 100 to indicate which share of the people (percentage) would follow vaccine recommendations.*	_____%

Section C: Past vaccinations

In the following questions we want to ask you about your vaccination status.		
C 01	Have you ever been vaccinated against HPV (Human Papilloma Virus)?	0 No, but I know what HPV is.

		1 No, and I have never heard of HPV.<br>2 Yes<br>97 I don't know.
C 02	Have you ever been vaccinated against Polio?	0 No, but I know Polio is.<br>1 No, and I have never heard of Polio.<br>2 Yes<br>97 I don't know.
C 03	Have you ever been vaccinated against Covid-19?	0 No, but I know what Covid-19 is.<br>1 No, and I have never heard of Covid-19.<br>2 Yes<br>97 I don't know.

Section D: vaccination confidence and vaccination intentions

**No.**	**Question**	**Code**
D 01	I worry about the side effects of vaccines.	0 Strongly disagree<br>1 Disagree<br>2 Somewhat disagree<br>3 Neither agree nor disagree<br>4 Somewhat agree<br>5 Agree<br>6 Strongly agree
D 02	After getting vaccinated, I feel protected.	0 Strongly disagree<br>1 Disagree<br>2 Somewhat disagree<br>3 Neither agree nor disagree<br>4 Somewhat agree<br>5 Agree<br>6 Strongly agree
D 03	I believe that vaccines often cause more harm than good.	0 Strongly disagree<br>1 Disagree<br>2 Somewhat disagree<br>3 Neither agree nor disagree<br>4 Somewhat agree<br>5 Agree<br>6 Strongly agree
D 04A	If Polio article: I intend to get vaccinated against Polio with the new vaccine or will encourage one of my family members to do so.	0 Strongly disagree<br>1 Disagree<br>2 Somewhat disagree<br>3 Neither agree nor disagree

		4 Somewhat agree<br>5 Agree<br>6 Strongly agree
D 04B	If HPV article: I intend to get vaccinated against HPV or will encourage one of my family members to do so.	0 Strongly disagree<br>1 Disagree<br>2 Somewhat disagree<br>3 Neither agree nor disagree<br>4 Somewhat agree<br>5 Agree<br>6 Strongly agree
D 04C	If COVID-19 article: I intend to get vaccinated against COVID-19 with the new vaccine or will encourage one of my family members to do so.	0 Strongly disagree<br>1 Disagree<br>2 Somewhat disagree<br>3 Neither agree nor disagree<br>4 Somewhat agree<br>5 Agree<br>6 Strongly agree

New polio eradication campaigns in Africa: Fighting a deadly disease with a new vaccine

Polio – a viral infection that can cause disabilities and death - has recently re-surfaced in many African countries. As a response many African countries such as Ghana, Kenya, Nigeria, and Uganda are currently rolling-out new widespread vaccine campaigns.

The upcoming vaccine campaigns plan to use a new vaccine called “nOPV2”. The World Health Organization (WHO) recently approved this vaccine which shall address a new variant of the polio virus. It will be the first time that the vaccine is used in Africa.

The new “nOPV2” polio vaccine was shown to be effective in preventing polio. Likewise, it is considered safe and does not provoke any mutations in humans. The World Health Organization (WHO) is very optimistic that the new vaccine will help eradicate polio worldwide. Make sure that you and your children get vaccinated!

An update on HPV vaccination campaigns across Africa: Cervical cancer about to get eliminated

HPV, short for Human Papilloma Virus, is a main cause of cervical cancer. Together with breast cancer it is the most common type of cancer among women in Africa. In an attempt to reduce the number of HPV cases in their countries, several African states such as Ghana, Nigeria, and Tanzania are providing HPV vaccines to a large share of their population.

Routinely, HPV vaccines are offered to teenage girls who are between 10 and 14 years old, though older girls and women can receive them too. These days many African countries are receiving two vaccines, called “GARDASIL 4” and “CERVARIX” that are already used in other countries, e.g. the U.S. and Germany. Over the next years many African countries plan to administer these two vaccines as part of their national HPV vaccination campaigns.

The two HPV vaccines “GARDASIL 4” and “CERVARIX” are proven to be very effective against HPV. They have been reliably used for years in many countries, such as the U.K., the U.S., and Germany. The vaccines contributed to a substantial decrease of cervical cancer cases in those countries. Make sure that you and your child get vaccinated against HPV to prevent cervical cancer!

Another COVID-19 vaccine: A new Omicron-adapted vaccine as game changer

Over the last three years COVID-19 led to more than 600 million infections and about 6 million deaths around the world. While COVID-19 started with the so called “Alpha” variant, the virus has mutated leading to some more infectious and deadly mutations. In Africa the most dominant form of COVID-19 is the so-called “Omicron” variant.

Recently, a new vaccine called “COMIRNATY” - developed by pharmaceutical giants Pfizer and BioNTech - got approved in the U.S. and most European countries. It aims to handle the “Omicron” variant more effectively. While this new vaccine is currently rolled out in Western countries, many African countries are expected to receive the vaccine soon via the World Health Organization’s (WHO) COVAX facility.

The WHO and countries such as the U.S. and Germany are very optimistic that the “COMIRNATY” vaccine will substantially reduce the risk of severe COVID-19 cases and deaths. In fact, scientific studies show that the new vaccine is quite effective in preventing severe “Omicron” cases. Make sure to get vaccinated against COVID-19 to protect yourself from illness and death!

Table B8. Correlation matrix of main variables

	Vac. hesitancy	Vac. confidence	Trust science	Trust pharma	Trust gov.	Trust media	Trust society
Vaccine hesitancy	1						
Vaccine confidence	-0.259***	1					
Trust in science	-0.087***	0.164***	1				
Trust in pharma	-0.127***	0.430***	0.112***	1			
Trust in government	-0.221***	0.383***	0.311***	0.318***	1		
Trust in media	-0.068***	0.112***	0.263***	0.091***	0.307***	1	
Trust in society	-0.137***	0.206***	0.063***	0.121***	0.266***	0.110***	1

Table B9. Balance table

Variable	COVID-19	Polio	HPV	Kruskal-Wallis test (p-value)
Age	29.01	29.05	29.39	.34
	(8.70)	(8.80)	(8.84)	
Female	0.32	0.37	0.37	.00
	(0.47)	(0.48)	(0.48)	
Married	0.33	0.32	0.32	.93
	(0.47)	(0.47)	(0.47)	
(Self-)employed	0.56	0.56	0.57	.72
	(0.50)	(0.50)	(0.50)	
Wealth	1.91	1.90	1.89	.47
	(0.59)	(0.62)	(0.60)	
No or primary education	0.02	0.02	0.02	.18
	(0.12)	(0.15)	(0.15)	
Secondary education	0.25	0.24	0.24	.77
	(0.43)	(0.43)	(0.43)	
Christian	0.86	0.88	0.87	.54
	(0.34)	(0.33)	(0.33)	
Muslim	0.10	0.08	0.09	.39
	(0.30)	(0.28)	(0.28)	
Other religion	0.04	0.04	0.04	.99
	(0.19)	(0.20)	(0.20)	
Agreeableness	2.18	2.17	2.08	.49
	(2.30)	(2.30)	(2.31)	
Openness	1.16	1.17	1.16	.96
	(2.07)	(1.99)	(2.00)	

Note: The table reports summary statistics of the experimental groups (sub-samples per vaccination type) and p-values of the Kruskal-Wallis rank test.
